# Long non-coding RNA ERICH3-AS1 is an unfavorable prognostic factor for gastric cancer

**DOI:** 10.7717/peerj.8050

**Published:** 2020-01-28

**Authors:** Qiongyun Chen, Xiaoqing Huang, Xuan Dong, Jingtong Wu, Fei Teng, Hongzhi Xu

**Affiliations:** 1 Department of Gastroenterology, Zhongshan Hospital, Xiamen University, Xiamen, Fujian, China; 2 Department of Chinese Tranditional Medicine, Zhongshan Hospital, Xiamen University, Xiamen, Fujian, China; 3 Department of Endocrinology, the First Affiliated Hospital of Xiamen University, Xiamen, China

**Keywords:** ERICH3-AS1, Gastric cancer, Prognosis, Tumorigenesis

## Abstract

Long non-coding RNAs (lncRNAs) play important roles in gastric cancer (GC), but the mechanism is not fully clear. ERICH3-AS1 (ERICH3 antisense RNA1) is affiliated with the non-coding RNA class which has proven to be involved in the prognostic of GC, but the function of ERICH3-AS1 is still unclear. In this study, we aim to explore the potential function of ERICH3-AS1 in the development of GC and analyze the prognostic role of ERICH3-AS1 in GC. We found that the lncRNA ERICH3-AS1 was significantly up-regulated in GC tissues in the analysis of The Cancer Genome Atlas (TCGA) data; the Kaplan-Meier analysis showed that the higher the expression of ERICH3-AS1 was, the earlier the recurrence and the poorer the prognosis would be in patients. Cox univariate and multivariate analyses revealed that ERICH3-AS1 was a risk factor of disease-free survival (DFS) (*p* < 0.05) and overall survival (OS) (*p* < 0.05) of patients. Through Gene Ontology (GO) and Kyoto Encyclopedia of Genes and Genomes (KEGG) analyses, it demonstrated that the ERBB pathways, the mitogen-activated protein kinase (MAPK) pathways, the MTOR pathways, p53 pathways and Wnt pathways were differentially enriched in ERICH3-AS1 high expression phenotype. Furthermore, the correlation analysis showed that ERICH3-AS1 had significant correlations with apoptosis-related proteins such as BCL2L10 and CASP14; cell cycle-associated proteins CDK14 and invasion and migration-associated proteins such as MMP20, MMP26 and MMP27. In summary, we identified that increased ERICH3-AS1 might be a potential biomarker for diagnosis and independent prognostic factor of GC. Moreover, ERICH3-AS1 might participate in the oncogenesis and development of tumors via cell cycle and apoptosis pathway mediated by ERBB, MAPK, MTOR, p53 and Wnt pathways.

## Background

Gastric cancer (GC) is one of the common malignant tumors of the digestive system, and has high incidence and mortality rates in China. According to the cancer statistical data in 2015 (Chen et al., 2016b; Siegel, Naishadham & Jemal, 2013; Sugano, 2015), both morbidity and mortality rates of gastric cancer rank second in malignant tumors in China, seriously threatening people’s health. But the occurrence and development mechanism of gastric cancer has not been fully elucidated (Hunt et al., 2015). The prognosis of GC is mainly dependent on tumor stage, location, histological type and therapy. Patients with early tumors had a better prognosis (Song et al., 2017). However, the early diagnosis rate of gastric cancer in China is still low. It is necessary to identify reliable predictors that are related to tumor stage and prognosis, and provide new targets for diagnosis, treatments and prognostic evaluation. Although CEA, CA724, CA19-9 are considered to be biomarkers in clinical practice, the sensitivity and specificity are not reliable (Wada et al., 2017). More effective tumor molecules are needed to distinguish the oncogenesis and development of tumors.

In recent years, the regulating effect of non-coding RNA gene expression has attracted more and more attention (Djebali et al., 2012). Long non-coding RNA (lncRNA) is a kind of RNA molecule with more than 200 bp in length without the ability to encode proteins. Although it does not have the ability to encode proteins, lncRNA could regulate the gene expression at transcription level, post-transcri- ptional level and translation level. More and more studies have shown that lncRNA plays an important role in the occurrence, development and prognosis of tumors (Fan et al., 2016; Fei et al., 2016; Gu et al., 2015). In gastric cancer (GC), according to reports, there are many lncRNAs involved in cell migration, invasion and proliferation (MacLeod et al., 2017; Li et al., 2019; Ma et al., 2019; Hu, Wang & Li, 2019), and even participated in 5-fluorouracil resistance via increasing Bcl2 expression (Du et al., 2019). For example, lncRNA PVT1 could directly bind FOXM1 protein and increased FOXM1 pasttranslationally leading to cell proliferation and invasion in vitro and in vivo (Xu et al., 2017). Some other lncRNAs such as GAS5, MEG3, GASLL1 promoted cell proliferation or metastasis via MTOR, p53 and Wnt pathway (Shen et al., 2019; Wei & Wang, 2017; Peng et al., 2019). With the deep-going study of lncRNA, more and more evidence reveals that the function of lncRNA is no less than the importance of encoding protein and even plays an important role in the proliferation and migration of tumors.

ERICH3-AS1, also named as ERICH3 antisense RNA1, is an RNA gene and affiliated with the non-coding RNA class, located in chr1: 74,577,430–74,626,098 (GRCh38/hg38). Little is known about the function and role of ERICH3-AS1 in tumors. It was reported previously that ERICH1-AS1 could predict the prognosis of patients with non-small cell lung cancer and may be a potential biomarker of non-small cell lung cancer (Tang et al., 2015). As a member of the same family, so far a few studies about the relationship between ERICH3-AS1 and GC have been reported. Last year, an article proved that ERICH3-AS1 might involve in GC (HR 1.541, 95%CI [1.108–2.145], *p*-value 0.01) (Zhu et al., 2018). However, the function of ERICH3-AS1 in GC is still unclear. Therefore, in this study, we aim to explore the potential function of ERICH3-AS1 in the oncogenesis and development of GC and analyze the prognostic role of ERICH3-AS1 in GC based on The Cancer Genome Atlas (TCGA).

## Material and Methods

### Data collection

The RNA-Seq raw count data were download the from TCGA database (https://tcga-data.nci.nih.gov/tcga/). In addition, the clinical data including age, gender, grade, TNM stage, overall survival time, disease free time and status of these patients were also downloaded. At last, in total of 3,337 gene expression file which include 32 normal tissue and 375 tumor samples was significantly changed in tumor tissues (fold change = 1, *p* adj = 0.05). In the clinical data, 373 patients include overall survival data and status, and 303 patients include disease free time and status.

### An analysis on the relationship between ERICH3-AS1 and clinicopathologic parameters

In order to calculated the relationship between the expression level of ERICH3-AS1 and clinicopathologic parameters. The patients were sorted from low to high according to the expression of ERICH3-AS1. Then, they were divided into high-expression group (>median of ERICH3-AS1) and low-expression group (≤ median of ERICH3-AS1).

### Gene set enrichment analysis (GSEA)

The GSEA 2.2.3 software was used for analysis. Data sets (c2.cp.kegg.v5.2.symbols.gmt and c5.bp.v5.2.symbols.gmt) were obtained from molecular signatures database (MsigDB) on the GSEA website (http://software.broadinstitute.org/gsea/downloads.jsp). In this study, GSEA was used to elucidate the significant survival difference between high- and low- ERICH3-AS1 groups. Then enrichment analysis was performed by statistical methods of default-weighted enrichment. The number of random combination was set at 1,000 times. The expression of ERICH3-AS1 was used as a phenotype label. The pathway enrichment was analyzed based on nominal *p* value and normalized enrichment score (NES).

### Statistical methods

Statistical analysis was performed by Statistical Product and Service Solutions (SPSS 22.0). Edger R package was used to calculate the difference between normal tissue and tumor samples in TCGA datasets. *χ*^2^ test and Fisher’s exact probability test were used for the comparison of correlation analysis between ERICH3-AS1 and clinicopathological parameters. The Kaplan–Meier (K-M) and Log-rank tests were adopted for survival analysis. Univariate and multivariate Cox proportional hazard models were used to compare relative risk and analyze the influences of each factor on the survival rate. Cor package was used to calculate pearson correlation of the all gene with ERICH3-AS1. *p* < 0.05 indicated that the differences had statistical significance.

## Results

### ERICH3-AS1 was significantly up-regulated in gastric cancer tissues

**Figure 1:**
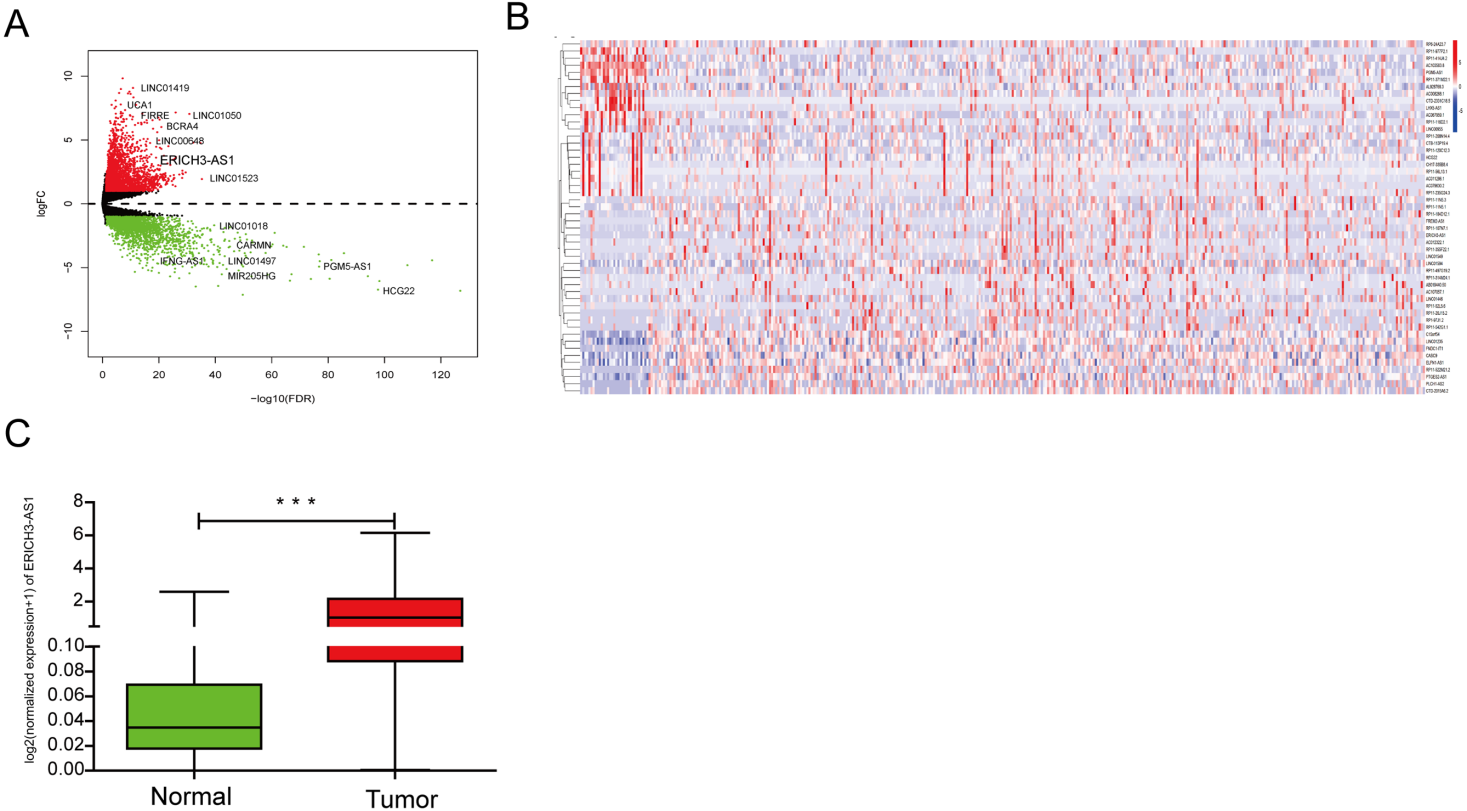
The analysis result of TCGA database. (A) Volcano map of differentially expressed lncRNAs in gastric cancer in TCGA. (B) Dysregulated lncRNAs in gastric cancer in TCGA. (C) ERICH3-AS1 was up-regulated in gastric cancer in TCGA.

The lncRNA expression profiles was detected from TCGA database, indicating that 2608 upregulated and 729 downregulated lncRNA form C and normal mucosal tissues (Figs. 1A and 1B). The analysis of TCGA database showed that compared with that in normal gastric mucosal tissues, the ERICH3-AS1 expression in GC was significantly increased (Fig. 1C).

### Relationship between ERICH3-AS1 expression and clinical data

In order to study the relationship between ERICH3-AS1 expression and clinical data, the patients were divided into high-expression group and low-expression group based on the median of ERICH3-AS1 expression level. As shown in Table 1, the higher the ERICH3-AS1 expression was, the higher the tumor residual rate would be and the higher the tumor metastasis rate would also be. However, the expression level of ERICH3-AS1 was not related to age, tumor lymph nodes metastasis (TNM) stage, gender, depth of tumor invasion, tumor differentiation and lymph node metastasis (Table 1).

**Table 1:** Association between ERICH3-AS1 expression and clinicopathological characteristics of patients with gastric cancer (*n* = 368).

Clinicopathologic features	Number of cases	ERICH3-AS1 expression		x2-test
		Low (*n* = 184)	High (*n* = 184)	*p* value
Age(Years)				
≤60	121	60	61	0.912
>60	247	124	123	
Residual tumor				
Absent	311	163	148	0.040^*^
Present	56	21	35	
TNM stage				
I+II	170	78	92	0.143
III+IV	198	106	92	
Gender				
Male	235	114	121	0.448
Female	133	70	63	
Depth of invasion				
T1+T2	99	44	55	0.196
T3+T4	269	140	129	
Distant metastasis				
No	334	174	160	0.012^*^
Yes	34	10	24	
Histological differentiation				
Well+moderate	143	67	76	0.335
Poor+undifferentiated	225	117	108	
Lymph node metastasis				
Absent	109	46	63	0.0522
Present	259	138	121	

**Notes.**

* *p* < 0.05.

### Role of ERICH3-AS1 in GC patient survival

**Figure 2:**
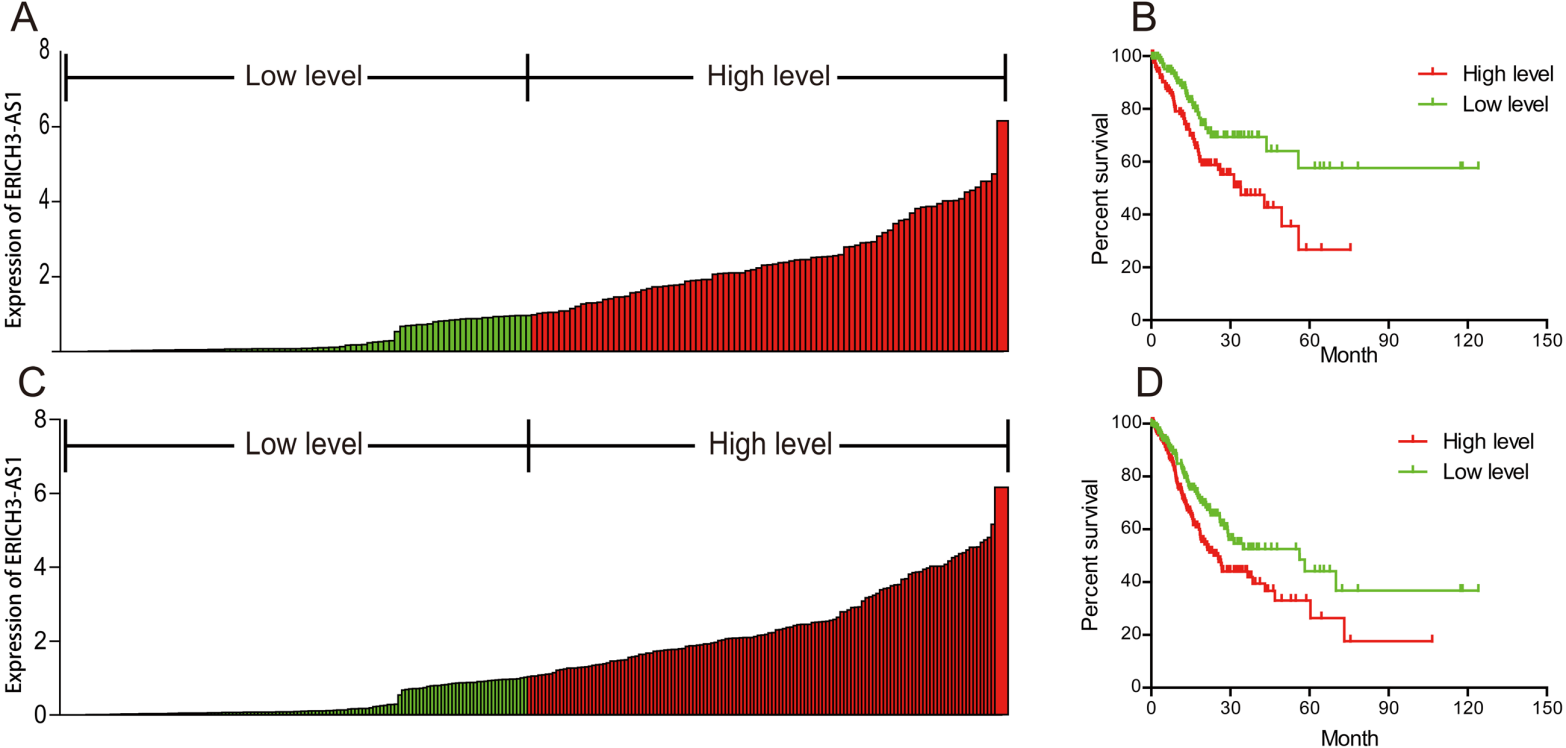
ERICH3-AS1 expression was negatively correlated with disease-free survival (DFS) and overall survival (OS) of patients. (A) Expression distribution of ERICH3-AS1 in cox model of DFS. (B) Kaplan-Meier curves for DFS time. (C) Expression distribution of ERICH3-AS1 in cox model of OS. (D) Kaplan-Meier curves for OS time.

To investigate the relationship between ERICH3-AS1 and prognosis of patients, Kaplan–Meier analysis and log-rank test were performed to analyze the relationship of ERICH3-AS1 expression with DFS and OS of patients. The results showed that the higher the expression of ERICH3-AS1 was, the earlier the recurrence (Figs. 2A and 2B), the shorter the OS (Figs. 2C and 2D) and the poorer the prognosis would be. Later, univariate Cox analysis was performed for DFS of patients, and the results showed that gender, size of residual tumor, lymph node metastasis and ERICH3-AS1 expression level were risk factors of DFS; then multivariable Cox analysis was performed, and it was obtained that the size of residual tumor and ERICH3-AS1 expression level were independent risk factors of DFS (Table 2). Finally, univariate Cox analysis was performed for OS of patients, and the results showed that the gender, tumor differentiation, size of residual tumor, tumor staging, distant metastasis, lymph node metastasis and ERICH3-AS1 expression level were risk factors of prognosis; then multivariable Cox analysis was performed, and it was obtained that the gender, tumor differentiation, size of residual tumor, tumor staging and ERICH3-AS1 expression level were independent risk factors of prognosis (Table 3). The above data indicated ERICH3-AS1 was a prognostic factor in GC and a higher expression of ERICH3-AS1 was negatively correlated with poor disease-free survival (DFS) and overall survival (OS).

**Table 2:** Univariate and multivariate Cox regression analysis of overall survival in GC patients.

Variables	Univariate analysis			Multivariate analysis		
	*P* value	HR	95% CI	*P* value	HR	95% CI
Age (≤60 vs. >60)	0.007	1.023	(1.006–1.040)	<0.000	1.037	(1.019–1.055)
Gender (Female vs. Male)	0.144	0.767	(0.537–1.095)	0.42	0.861	(0.598–1.239)
Tumor grade (G1 vs. G2 vs. G3)	0.011	1.466	(1.090–1.971)	<0.000	1.763	(1.294–2.402)
Residual tumor (No vs. Yes)	<0.000	1.785	(1.512–2.107)	<0.000	1.776	(1.472–2.143)
Stage (Stage I vs. Stage II vs. Stage III vs. Stage IV)	<0.000	1.656	(1.345–2.038)	<0.000	1.484	(1.202–1.832)
Distant metastasis (age ≤60) (No vs. Yes)	0.001	3.481	(1.675–7.235)	0.653	1.086	(0.758–1.554)
Distant metastasis (age>60) (No vs. Yes)	0.296	1.217	(0.842–1.759)			
Lymph node metastasis (age ≤60) (N0 vs. N1 vs. N2 vs. N3)	0.036	1.534	(1.029–2.287)	0.28	1.146	(0.895–1.467)
Lymph node metastasis (age>60) (N0 vs. N1 vs. N2 vs. N3)	0.001	1.475	(1.172–1.855)			
Depth of invasion (age ≤60) (T1 vs. T2 vs. T3 vs. T4)	0.344	1.211	(0.815–1.799)	0.65	1.058	(0.829–1.351)
Depth of invasion (age>60)(T1 vs. T2 vs. T3 vs. T4)	<0.000	1.542	(0.214–1.960)			
ERICH3-AS1 level (Low vs. High)	0.001	1.211	(1.080–1.358)	<0.000	1.256	(1.117–1.411)

**Notes.**

CI confidence interval HR hazard ratio

**Table 3:** Univariate and multivariate Cox regression analysis of disease-free survival in GC patients.

Variables	Univariate analysis			Multivariate analysis		
	*P* value	HR	95%	*P* value	HR	95%
Age (≤60 vs. >60)	0.525	0.993	0.972–1.015	0.973	1.000	0.976–1.024
Gender (Female vs. Male)	0.001	2.441	1.418–4.203	0.013	2.016	1.160–3.502
Tumor grade (G1 vs. G2 vs. G3)	0.319	1.265	0.797–2.008	0.454	1.201	0.744–1.938
Residual tumor (No vs. Yes)	<0.000	4.063	2.019–8.175	0.002	3.065	1.514–6.204
Stage-Female (Stage I vs. Stage II vs. Stage III vs. Stage IV)	0.029	2.224	1.087–4.547	0.486	1.185	0.735–1.912
Stage-Male (Stage I vs. Stage II vs. Stage III vs. Stage IV)	0.062	1.366	0.984–1.897			
Distant metastasis (No vs. Yes)	0.521	1.477	0.449–4.858	0.764	1.225	0.325–4.618
Lymph node metastasis (N0 vs. N1 vs. N2 vs. N3)	<0.000	1.480	1.200–1.825	0.002	1.406	1.137–1.738
Depth of invasion (T1 vs. T2 vs. T3 vs. T4)	0.503	1.105	0.826–1.478	0.578	0.914	0.666–1.255
ERICH3-AS1 level (Low vs. High)	0.017	1.206	1.035–1.406	0.006	1.243	1.063–1.452

**Notes.**

CI confidence interval HR hazard ratio

### ERICH3-AS1-related signaling pathways based on GSEA

**Figure 3:**
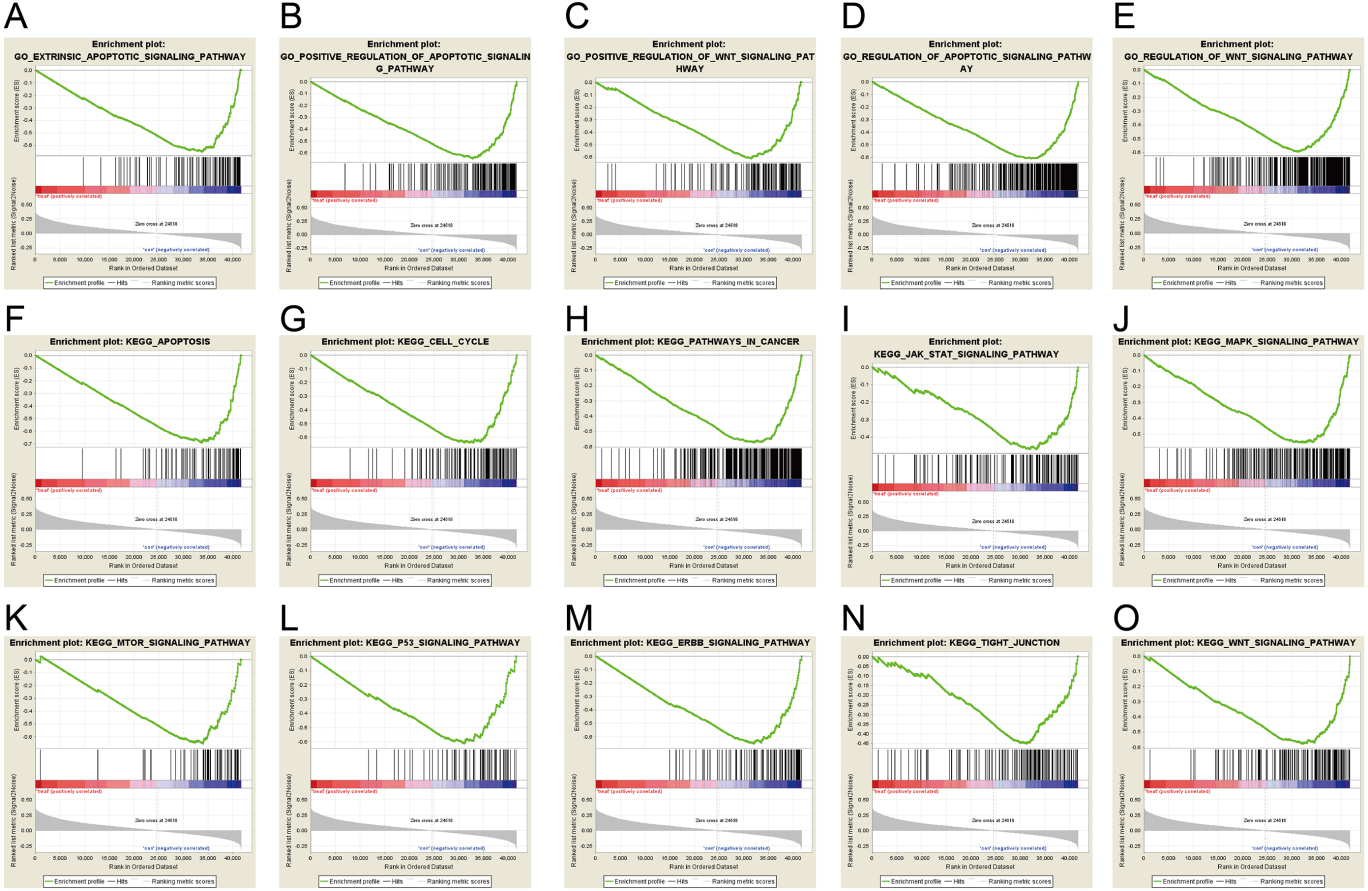
ERICH3-AS1-related signaling pathways based on GSEA. The GSEA with c5.bp.v6.1.symbols.gmt motif of the high and lowERICH3-AS1 expression groups identified extrinsic the apoptotic signaling pathway (A), the positive regulation of apoptotic signaling pathway (B), the positive of Wnt signaling pathway (C), the regulation of apoptotic signaling pathway (D), the regulation of Wnt signaling pathway (E). The GSEA with c2.cp.kegg.v6.1.symbols.gmt motif of the high and lowERICH3-AS1 expression groups identified apoptosis (F), cell cycle (G), pathways in cancer (H), Jak STAT signaling pathway (I), the MAPK signaling pathway (J), the MTOR signaling pathway (K), the P53 signaling pathway (L), the ERBB signaling pathway (M), the tight junction (N), and the Wnt signaling pathway (O).

Gene set enrichment analysis (GSEA) was used to investigate signaling pathway involved in GC and demonstrated significant difference (NOM *p*-value <0.05) in enrichment of MSigDB Collection. Based on the median of ERICH3-AS1 expression, the patients were divided into high-expression group and low-expression group. Through Gene Ontology (GO) and Kyoto Encyclopedia of Genes and Genomes (KEGG) analysis, it was obtained that ERICH3-AS1 was mainly enriched in the cell cycle and apoptosis, which may affect cell cycle and apoptosis through ERBB, mitogen-activated protein kinase (MAPK), mammalian target of rapamycin (MTOR), p53 and Wnt pathways (Fig. 3).

**Figure 4:**
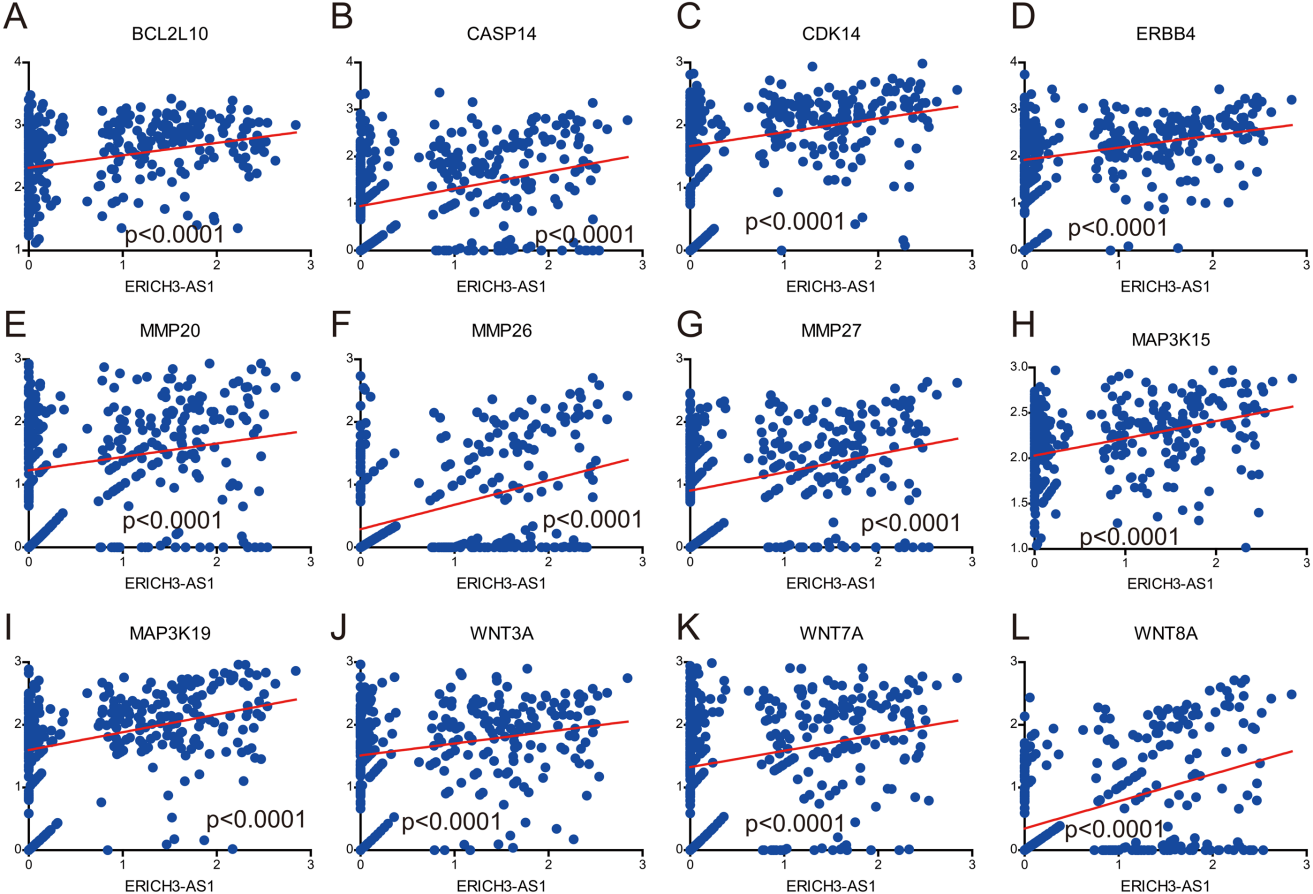
Correlation analysis between expression of ERICH3-AS1 and key cellular molecules. (A) Correlation analysis between expression of ERICH3-AS1 and BCL2L10. (B) Correlation analysis between expression of ERICH3-AS1 and CASP14. (C) Correlation analysis between expression of ERICH3-AS1 and CDK14. (D) Correlation analysis between expression of ERICH3-AS1 and ERBB4. (E) Correlation analysis between expression of ERICH3-AS1 and MMP20. (F) Correlation analysis between expression of ERICH3-AS1 and MMP26. (G) Correlation analysis between expression of ERICH3-AS1 and MMP27. (H) Correlation analysis between expression of ERICH3-AS1 and MAP3K15. (I) Correlation analysis between expression of ERICH3-AS1 and MAP3K19. (J) Correlation analysis between expression of ERICH3-AS1 and WNT3A. (K) Correlation analysis between expression of ERICH3-AS1 and WNT7A (L) Correlation analysis between expression of ERICH3-AS1 and WNT8A.

It was obtained that apoptosis-related proteins, Bcl-2-like protein 10 (BCL2L10) and Caspase 14 (CASP14) (Figs. 4A and 4B), cell cycle-associated proteins, CDK14 (cyclin-dependent kinase 14) (Fig. 4C), and invasion and migration-associated proteins, matrix metalloproteinase 20 (MMP20), MMP26 and MMP27 (Figs. 4E, 4F and 4G) were positively correlated, and positively correlated with MAPK, Wnt and ERBB pathway (Figs. 4D, 4H, 4I, 4J, 4K and 4L). These data revealed ERICH3-AS1 induced the cellular molecules such as BCL2L10, CDK14, MMPs to regulate cell cycle and apoptosis in GC, providing new targets for diagnosis, treatments and prognostic evaluation.

## Discussion

The incidence rate of gastric cancer ranks first in the digestive system malignant tumors in China. According to the survey data of World Health Organization, the morbidity and mortality rates of gastric cancer in Chinese patients is twice that of the average in the world. Invasion and metastasis of gastric cancer cells are the causes of death in most patients with gastric cancer (Chen et al., 2016a). The molecular mechanism of gastric cancer has always been a research hotspot of tumor over the years.

In the whole genome, the number of lncRNA accounts for 99% of the total human gene transcripts, and it is almost involved in all epigenetic regulation as a “man behind the curtain”, suggesting that lncRNA plays an important role in the complex life activities, including various diseases, of advanced eukaryotes (Shang et al., 2016; Zhang et al., 2016). Compared with the protein-coding gene, lncRNA has obvious developmental stage-specificity and tissue specificity (Hu et al., 2015). The function and mechanism of lncRNA are diverse; more importantly, lncRNA can regulate the expressions of protein-coding genes from multiple levels and multiple perspectives (Dang et al., 2015; Ding et al., 2015).

In our study, it was found in the analysis of TCGA data based on high throughput RNA sequencing that the lncRNA ERICH3-AS1 was significantly up-regulated in gastric cancer tissues compared with normal tissues; and the Kaplan–Meier analysis showed the higher the expression of ERICH3-AS1 was, the earlier the recurrence and the poorer the prognosis would be in patients. Cox univariate and multivariate analyses revealed that ERICH3-AS1 was a risk factor of DFS and OS of GC. Through GO and KEGG analyses, it was found that ERICH3-AS1 mainly induced apoptosis-related proteins, cell cycle-associated proteins and invasion and migration-associated proteins to regulate the cell cycle, apoptosis and Wnt, p53, mTOR, janus kinase-signal transducer and activator of transcription (JAK-STAT) and MAPK pathways. It revealed the potential mechanism of ERICH3-AS1 in the oncogenesis and development of GC by participating in cell cycle, apoptosis and invasion. However, more investigations such as cell transfection, cell proliferation assay, colony formation assay, cell cycle assay, transwell cell migration and invasion assay, qRT-PCR and western blot need to be carried out to validate the function and regulation between ERICH3-AS1 and related genes in GC.

In this study, the clinical significance of ERICH3-AS1 in GC and its possible biological mechanism were reported for the first time, providing a theoretical basis for the treatment of gastric cancer.

## Conclusion

In summary, we identified that ERICH3-AS1 was highly expressed in patients with GC and was an independent prognostic factor of GC. We discovered that the expression level of ERICH3-AS1 was negatively correlated with DFS and OS of patients and GSEA analysis confirmed that gene sets involved in cell cycle and apoptosis were significantly enriched in GC patients with higher levels of ERICH3-AS1.

## Additional Information and Declarations

### Data Availability

The following information was supplied regarding data availability: The RNA-Seq raw count data of gastric cancer were downloaded from the TCGA database (https://portal.gdc.cancer.gov/) using the search term “TCGA-STAD”.
